# 

**Published:** 1853-07

**Authors:** 


### CONTENTS.

### JOURNAL.

Page.

ARTICLE I.—Odontalgia. Its Pathology and Treatment. By J. P. Togg, M. D. . . . . . . . . . . . . 513
ARTICLE II.—Dental Patents. By Robert Arthur, M. D., D. D. S. 530
ARTICLE III.—Gold, Gold-beating, Compounds and Alloys. By Prof. R. N. Wright, M. D. . . . . . . . . 551
ARTICLE IV.—On the Morbid Changes of Saliva. By Prof. A. Snowden Piggot, . . . . . . . . . . 565
ARTICLE V.—A Case of Reproduction of the Inferior Maxillary Bone. By Wm. Jones, M. D., Kenton, Ohio. . . . . . 592
ARTICLE VI.—The Replacement of the Teeth. By C. F. Cushman, D. D. . . . . . . . . . . . 597
ARTICLE VII.—An Extraordinary Case of Hemorrhage. By T. C. Harris, Esq., London, . . . . . . . . . . 599
ARTICLE VIII.—An Exostosis on the Root of the First Left Lower Molar. By Edwin Kreonele, Hyde Park, London, . . 600

### SELECTED ARTICLES.

ARTICLE IX.—On the Physiology of Absorption and of Respiration. By M. C. Bernard, . . . . . . . . . . 602
ARTICLE X.—Removal of the Superior Maxilla on the left side, with a large Tumor involving that Bone. Under the care of Mr. Hancock, . . . . . . . . . . . . . 629
ARTICLE XI.—A Counterblast against Tobacco, . . . . . 632
ARTICLE XII.—Disease of the Antrum produced by a Fall, . 635
ARTICLE XIII.—Effects of Chloroform on the Blood, . . . . 637
ARTICLE XIV.—Excision of the Inferior Maxillary Bone for Caries. By W. G. Bullock, M. D., of Savannah, Geo. . . . 640
ARTICLE XV.—Patent Crystalline Sponge Gold, . . . . . 642
ARTICLE XVI.—Extraction of Teeth. By Dr. J. Taylor, . . 645

### BIBLIOGRAPHICAL.

The Philosophy of Medical Science, Boylston's Prize Essay, 1849, 649
Headland on the Action of Medicines in the system, . . . 653

*Contents.*

Page.
Respiration subservient to Nutrition; a Thesis presented to the Medical Faculty of Harvard University, March, 1850, . 656
A Clinical Phrase Book, in English and German, . . . . 656
Treatise on Uterine Displacement, . . . . . . . . . 657
A Treatise on Rational Pathology, . . . . . . . . 657
A Treatise on Dentistry, . . . . . . . . . . 658
Ancora su la Dentizione della Sapienza, . . , . . 659
A Practical Treatise on the Diseases of Children, . . . . 660

### EDITORIAL DEPARTMENT.

Chemistry as a branch of Medical Education, . . . . . 661
Singular Development of an Artery of the Gums, . . . . . 664
Editorial Change, . . . . . . . . . . . . 664
Omitted, . . . . . . . . . . . . . . . . 664
The End of the Volume, . . . . . . . . . . 664

